# Micafungin-induced rhabdomyolysis and myocardial injury: a case report

**DOI:** 10.3389/fmed.2025.1708395

**Published:** 2025-12-04

**Authors:** Qing Liu, Qin Li

**Affiliations:** Department of Respiratory and Critical Care Medicine, The First People’s Hospital of Lianyungang, Affiliated Hospital of Xuzhou Medical University, Lianyungang, Jiangsu, China

**Keywords:** micafungin, rhabdomyolysis, myocardial injury, adverse drug reaction, antifungal agents

## Abstract

An 82-year-old woman with pulmonary aspergillosis developed dark-colored urine, markedly elevated creatine kinase (3,125 U/L) and myoglobin (3884.4 ng/mL), and delayed myocardial injury (high-sensitivity troponin I: 26301.6 pg/mL) on day 2 after intravenous micafungin (200 mg/day) initiation. Symptoms and biomarkers resolved upon discontinuation, suggesting micafungin-induced rhabdomyolysis and myocardial injury. This first-reported case highlights the need for vigilance in elderly, multimorbid patients receiving polypharmacy, expanding understanding of micafungin’s safety profile.

## 1 Introduction

The incidence of invasive pulmonary aspergillosis has been progressively increasing due to widespread use of immunosuppressive agents, chemotherapy-induced neutropenia, and the growing number of hematopoietic stem cell and solid organ transplants (1). Currently, clinical antifungal regimens primarily consist of three major classes: triazoles (e.g., voriconazole, isavuconazole), echinocandins (e.g., micafungin, caspofungin), and polyenes (e.g., liposomal amphotericin B). Echinocandins exhibit fungicidal activity against *Candida* species while demonstrating fungistatic effects against *Aspergillus*. As one of the most commonly prescribed echinocandins, micafungin serves as first-line therapy for candidiasis and is also employed in invasive pulmonary aspergillosis (2). Its favorable safety profile and tolerability have made micafungin widely used in clinical antifungal therapy.

Rhabdomyolysis (RM) represents a clinical syndrome characterized by skeletal muscle cell necrosis and subsequent release of intracellular contents (including myoglobin, creatine kinase, and potassium) into systemic circulation, which may lead to severe complications such as electrolyte disturbances, acute kidney injury, and multi-organ failure (3). The diagnostic triad of RM (muscle pain, weakness, and dark urine) is observed in fewer than 10% of patients (4). Etiologies of RM are diverse and include trauma, strenuous exercise, electrolyte imbalances, genetic disorders, infections, and toxins. Numerous medications, particularly statins, antipsychotics, and certain antibiotics, are well-established causes of drug-induced rhabdomyolysis (DIR) (5). Although RM arises from diverse causes, the pathophysiology of DIR primarily involves sarcolemmal disruption and ATP depletion, leading to calcium influx into myocytes. This results in sustained muscle contraction, energy exhaustion, and activation of calcium-dependent proteases, ultimately causing myocyte lysis and the release of intracellular components (4, 5). Herein, we present a case of micafungin-induced RM and myocardial injury following intravenous administration.

## 2 Case presentation

An 82-year-old female with a 40-year history of hypertension and 6-year history of type 2 diabetes mellitus (managed with nifedipine sustained-release tablets, saxagliptin, and repaglinide) was admitted to the Respiratory and Critical Care Intensive Care Unit (ICU) on March 25, 2025, presenting with a 6-day history of productive cough, fever, and dyspnea. Her medical history included paroxysmal atrial fibrillation and cerebral infarction 5 years prior with residual right-sided hemiparesis (on maintenance aspirin and rosuvastatin therapy), as well as prior myomectomy. No known drug allergies were reported. Physical examination revealed a temperature of 38.4 °C, blood pressure of 151/85 mmHg, and delirious mental status with bilateral equal and sluggishly reactive pupils. Respiratory examination demonstrated diminished breath sounds in the left lung, coarse breath sounds in the right lung, and bilateral crackles without pleural friction rub. Cardiovascular assessment showed atrial fibrillation at 121 beats per minute without audible murmurs. Peripheral edema was noted with uncooperative muscle strength testing but normal muscle tone.

Initial laboratory investigations demonstrated type I respiratory failure, moderate anemia, hyperglycemia, hypoalbuminemia, and hypocalcemia. All the blood investigations are reported in Table 1. Chest CT revealed left lower lobe consolidation, bilateral pleural effusions, and atherosclerotic changes in the aorta and coronary arteries. Electrocardiography confirmed atrial fibrillation, while echocardiography showed left atrial enlargement, degenerative aortic valve with mild regurgitation and mild stenosis, and trivial mitral and tricuspid regurgitation. Lower extremity venous ultrasound identified right gastrocnemius muscular vein thrombosis. The patient was initiated on high-flow nasal cannula oxygen therapy (FiO₂ 50%, flow rate 40 L/min) and empirical biapenem for severe pulmonary infection. Persistent fever and respiratory symptoms prompted bronchoscopy on March 27, with Targeted next-generation sequencing (tNGS) of bronchoalveolar lavage fluid revealing Aspergillus flavus complex (169 reads), *Aspergillus nidulans* (34 reads), *Candida albicans* (34 reads), *Ureaplasma parvum* (24 reads), and Epstein–Barr virus (25 reads), establishing the diagnosis of pulmonary aspergillosis. Intravenous micafungin (200 mg daily) was commenced on March 30, resulting in defervescence and resolution of respiratory symptoms. Concurrent medications included metoprolol tartrate, rosuvastatin calcium, levothyroxine sodium, ambroxol hydrochloride, somatostatin, omeprazole, sivelestat sodium, human albumin, and insulin glargine. The clinical course was complicated by gastrointestinal bleeding manifested as melena and positive fecal occult blood testing, with hemoglobin declining. This was managed with proton pump inhibitor therapy, somatostatin infusion, blood transfusion, and parenteral nutrition.

**Table 1:** Laboratory investigations from day 1 to 16 of hospitalization.

Laboratory parameter	Day 1 (March 25)	Day 3 (March 27)	Day 5 (March 29)	Day 7 (March 31)	Day 10 (April 3)	Day 11 (April 4)	Day 14 (April 7)	Day 16 (April 9)
Leucocytes (10^9/L)	6.88	10.73	10.15	8.25	6.60	6.59	2.82	3.11
Erythrocytes (10^12/L)	3.06	2.46	2.33	2.79	2.98	2.80	2.58	2.81
Hemoglobin (g/L)	87	71	69	83	82	84	78	85
Platelets (10^9/L)	244	151	117	80	87	93	95	110
Neutrophils (%)	94.7	85.7	89.0	87.5	87.6	83.0	62.3	55.2
Lymphocytes (%)	4.6	9.8	7.8	8.1	7.8	12.1	28.2	32.9
Monocytes (%)	0.7	4.1	2.5	3.4	2.4	4.3	7.2	8.1
Eosinophils (%)	0.0	0.4	0.7	1.0	0.3	0.5	2.0	3.5
CRP (mg/L)	42	20	18	14	11	9	3	2
Procalcitonin (ng/mL)	0.18	0.12						
BDG assay (pg/mL)		<10						
GM assay (μg/L)		0.13						
Sodium (mmol/L)	146	155	149	143	141	137	142	141
Potassium (mmol/L)	4.8	4.0	4.0	3.2	3.6	3.8	3.6	3.8
Chloride (mmol/L)	115	120	115	105	99	103	106	106
Calcium (mmol/L)	1.8	1.9	1.9	1.8	1.9	2.0	2.0	2.0
Total bilirubin (μmol/L)		8.7		14.1	27.9	19.4		
Direct bilirubin (μmol/L)		2.5		4.0	8.3	5.1		
ALT (U/L)		30		25	83	82		
AST (U/L)		45		70	156	148		
ALP (U/L)		48		73	108	98		
Total protein (g/L)		42.0		43.4	53.2	47.8		
Albumin (g/L)		25.1		25.9	34.1	29.1		
Globulin (g/L)		16.9		17.5	19.1	18.7		
CK (U/L)	57	77		3,087	3,125	2,125	100	48
CK-MB (ng/mL)	1.06	1.83		4.36	67.40	65.05	3.59	2.90
hs-cTnI (pg/mL)	18	23		13	28	26,302	1,537	528
MYO (ng/mL)	89.7	208.5		3245.9	3884.4	2424.4	182.8	101.9
BNP (pg/mL)	206	131		414		106	161	
BUN (mmol/L)	18.5	16.0	11.6	6.0	5.4	7.6	6.9	
Creatinine (μmol/L)	56.6	60.2	65.1	52.8	47.5	31.6	28.8	
eGFR (mL/min/1.73m^2^)	75	71	65	80	89	134	147	
FPG (mmol/L)	21.6	8.8	8.3	8.8	8.2		7.3	
Triglycerides (mmol/L)		1.22						
Oxygenation Index	122	89	187	228	235		275	
PaCO₂ (mmHg)	34	41	43	43	46		43	
PT (s)	13.1	12.5	12.0	11.9	11.9	10.8	11.2	10.6
APTT (s)	33.5	30.3	27.0	25.7	27.3	27.4	30.2	30.2
INR	1.15	1.14	1.05	1.04	1.04	0.95	0.98	0.96

CRP, C-reactive protein; BDG, (1,3)-β-D-glucan; GM, Galactomannan; ALT, Alanine Aminotransferase; AST, Aspartate Aminotransferase; ALP, Alkaline Phosphatase; CK, creatine kinase; CK-MB, Creatinine Kinase—Muscle Brain; hs-cTnI, high-sensitivity cardiac troponin I; MYO, myoglobin; BNP, B-type Natriuretic Peptide; BUN, Blood Urea Nitrogen; eGFR, estimated Glomerular Filtration Rate; FPG, fasting plasma glucose; PT, Prothrombin Time; APTT, Activated Partial Thromboplastin Time; INR, International Normalized Ratio.

On March 31, the patient developed dark-colored urine with elevated urinary urobilinogen and occult blood, accompanied by markedly increased creatine kinase (CK), Creatinine Kinase-Muscle Brain (CK-MB) and myoglobin (MYO). RM was suspected, prompting discontinuation of rosuvastatin and sivelestat sodium with initiation of aggressive hydration. Despite stable renal function and improving oxygenation parameters, muscle enzymes continued to rise, peaking at CK 3125 U/L on April 3. Comprehensive evaluation including myositis antibody panel, autoimmune serology, and endocrine testing revealed no alternative explanations. The clinical findings strongly suggest DIR as the most probable diagnosis. Given the temporal association with micafungin initiation, the antifungal was discontinued on April 3. Subsequent laboratory monitoring demonstrated gradual decline in muscle enzymes, though a concurrent disproportionate elevation in high-sensitivity cardiac troponin I (hs-cTnI) was noted on April 4 without corresponding electrocardiographic or echocardiographic changes. Complete biochemical normalization was achieved by April 9 for muscle enzymes and April 16 for cardiac biomarkers. The patient showed no abnormal elevations in CK, troponin, or creatinine during follow-up. Following micafungin discontinuation, and in light of the patient’s improved body temperature and respiratory symptoms, stable pulmonary imaging findings, and continued improvement in infection-related biomarkers, no additional antifungal agents were administered. With ongoing supportive care including serial bedside bronchoscopy for secretion clearance and pathogen load reduction, there was no evidence of progression in pulmonary fungal infection. The patient was eventually discharged following a comprehensive course of rehabilitation.

## 3 Discussion

*Aspergillus fumigatus* remains the most prevalent causative pathogen of pulmonary aspergillosis (6). Due to its thermotolerant and xerotolerant characteristics, *Aspergillus flavus* has emerged as another common etiological agent, particularly in Asia, Africa, and the Middle East (7). Recent studies have demonstrated that the minimum inhibitory concentration (MIC) of polyenes against certain *A. flavus* strains exceeds 2 μg/mL, suggesting potential polyene resistance in some isolates (8). While voriconazole serves as the first-line treatment for pulmonary *A. flavus* infection, echinocandins may be considered as alternative monotherapy for patients with contraindications to triazoles. TNGS of bronchoalveolar lavage fluid from this patient identified *Aspergillus flavus* complex. Considering the patient’s advanced age, compromised baseline condition, and delirious state, micafungin was selected as the initial antifungal therapeutic agent.

Echinocandins function as selective inhibitors of 1,3-β-D-glucan synthase, effectively blocking the synthesis of this essential fungal cell wall component and causing osmotic instability leading to fungal cell lysis (9). Notably, 1,3-β-D-glucan also serves as a crucial pathogen-associated molecular pattern recognized by the Dectin-1 receptor on host immune cells. Experimental evidence demonstrates that caspofungin exposure induces dose-dependent increases in 1,3-β-D-glucan exposure on *A. fumigatus* cell walls (10). Furthermore, caspofungin-pretreated *A. fumigatus* hyphae exhibit enhanced susceptibility to polymorphonuclear neutrophil-mediated damage through upregulated Dectin-1 receptor expression. These findings suggest that the antifungal mechanisms of echinocandins extend beyond direct suppression of hyphal growth to include significant immunomodulatory effects on host defense responses (11).

Micafungin exhibits extensive plasma protein binding of up to 99%. The drug is primarily metabolized by arylsulfatase, catechol-O-methyltransferase, and several cytochrome P450 isoenzymes (including 3A4, 1A2, 2B6, and 2C), undergoing biotransformation into at least 11 identified metabolites (M1–M11) (12). The majority (>70%) of the drug is eliminated through fecal excretion, with minimal renal elimination. As human cells lack fungal cell walls, micafungin demonstrates limited host cytotoxicity. The most frequently reported adverse effects include infusion-related reactions, rash, fever, diarrhea, dyspnea, thrombocytopenia, and leukopenia. While studies suggested potential hepatocarcinogenicity in rats at high doses, clinical surveillance has not confirmed this risk in humans (12, 13). Although micafungin has been associated with polymorphic ventricular tachycardia (14), no previous cases of RM or myocardial injury have been documented.

The diagnosis of RM relies primarily on laboratory findings, including elevated CK levels, myoglobinuria, early-stage hypocalcemia, and renal dysfunction. CK represents the most sensitive diagnostic marker, typically rising within 12 h of muscle injury, peaking at 24–72 h, and normalizing within 5 days after cessation of injury. Current diagnostic criteria require CK levels ≥1,000 U/L or exceeding 5 times the upper limit of normal (15). Our patient developed dark-colored urine accompanied by markedly elevated CK and MYO levels (serum CK ≥ 1,000 U/L, exceeding 5-fold the upper limit of normal) on the second day following micafungin administration. Due to the patient’s impaired consciousness, clinical assessment of muscle weakness and myalgia was precluded. Although routine urinary myoglobin testing was unavailable in our laboratory, the subsequent decline in CK, CK-MB, and MYO levels following micafungin discontinuation, with complete normalization of CK by day 4 post-cessation, strongly supported the diagnosis. On the day of drug withdrawal, the patient exhibited a marked elevation in cardiac troponin levels that was disproportionate to the changes in CK and CK-MB. Neither electrocardiographic nor echocardiographic findings showed significant progression compared to previous examinations, thereby excluding acute myocardial infarction. Comprehensive evaluation excluded alternative etiologies including myositis, hyperparathyroidism, or myocardial infarction. While statins are common culprits of RM, this patient’s prolonged statin use without prior incidents and lack of improvement after statin discontinuation argued against this etiology. Other concurrent medications (biapenem, sivelestat sodium, somatostatin) showed no plausible association with the clinical presentation. The severity of infection is considered the pathophysiological basis for infection-associated RM (5). Viral and bacterial infections are well-established triggers of RM, with viral etiologies being more common. The underlying mechanisms may involve direct invasion of muscle tissue by pathogens, toxin-mediated damage, and immune-inflammatory responses (16). In contrast, clinical reports of fungal infection-induced RM remain scarce. Such cases predominantly affect severely immunocompromised hosts, such as patients with leukemia or organ transplantation, and are typically accompanied by refractory fungal sepsis (17). Although our patient had multiple comorbidities, she lacked significant immunosuppression. Moreover, the infection had clinically improved prior to the onset of RM, with resolution of both symptomatic and laboratory inflammatory parameters. The temporal relationship between micafungin discontinuation and rapid normalization of muscle enzyme profiles supports micafungin as the most probable etiology in this case.

Using the Naranjo Adverse Drug Reaction Probability Scale, the association between micafungin and RM/myocardial injury scored as “probable” (6 points): (1) temporal relationship with drug administration; (2) resolution after discontinuation; (3) exclusion of alternative causes; (4) objective laboratory confirmation. The Hartwig Severity Assessment Scale classified this as a level 4 (severe) adverse drug reaction (ADR), requiring ICU monitoring and prolonging hospitalization by >3 days. According to Siegel, Schumock and Thornton preventability scale, this ADR was deemed “not preventable” (18).

DIR represents a relatively uncommon clinical entity, with advanced age, renal insufficiency, and concomitant use of cytochrome P3A4 inhibitors identified as key risk factors (4). To our knowledge, this constitutes the first reported case of micafungin-induced RM with concurrent myocardial injury. The precise pathogenic mechanisms remain elusive due to limited existing research. The pathophysiology of RM fundamentally involves disruption of skeletal muscle membrane integrity, mitochondrial dysfunction, intracellular calcium overload, and consequent impairment of adenosine triphosphate (ATP) production (19). Current evidence suggests DIR may involve various degrees of mitochondrial dysfunction, including coenzyme Q10 depletion and mitochondrial permeability transition pore opening (20, 21). While micafungin cannot penetrate intact human cell membranes to directly affect mitochondria, *in vitro* studies by Hosler et al. (22) demonstrated that micafungin induces rapid swelling, rupture, and cytochrome C release in isolated mitochondria. Most reported DIR cases involve statin monotherapy or statin-drug interactions, though whether our case reflects a potential interaction between micafungin and rosuvastatin requires further investigation. The patient presented with significant cardiovascular risk factors that could predispose to myocardial injury. However, several lines of evidence support micafungin as the probable cause: (1) temporal association: both troponin levels and muscle enzyme profiles increased significantly following micafungin initiation and normalized synchronously after its withdrawal; (2) post-control onset: the myocardial injury occurred after resolution of fever and respiratory symptoms, with improvement in inflammatory markers (including C-reactive protein and interleukin-6) and imaging findings. This clinical course contrasts with fungal infection-related myocardial injury, which typically presents with persistent severe infection and high mortality; (3) exclusion of alternative acute etiologies: the patient remained hemodynamically stable without electrocardiographic or echocardiographic evidence of acute ischemia or infarction. Moreover, the delayed peak in high-sensitivity troponin (occurring 4 days after the rise in CK and MYO) was inconsistent with the typical evolutionary pattern of acute myocardial infarction. Compared to other antifungals like caspofungin or fluconazole, micafungin demonstrates minimal cardiotoxicity (23). However, Aisenberg et al. (24) identified seven DIR cases with myocardial injury through systematic database review, and autopsy studies of p-phenylenediamine poisoning victims have revealed RM-induced cardiogenic shock (25). We hypothesize that the observed myocardial injury may represent secondary damage following RM, with subsequent resolution paralleling RM improvement and thereby preventing catastrophic outcomes like cardiogenic shock.

The recommended adult dosage of micafungin ranges from 100 to 150 mg daily, potentially increased to 300 mg based on clinical indication. Studies confirm micafungin’s safety and tolerability even at doses up to 8 mg/kg/day, with no established dose-dependent relationship for hepatotoxicity or cardiotoxicity (26, 27). Our patient (height 160 cm, weight 70 kg) received treatment within this safe dosage range, suggesting no causal relationship between drug dosage and the observed adverse events.

This report describes an elderly female diagnosed with pulmonary aspergillosis who developed RM and myocardial injury on the second day following initiation of micafungin antifungal therapy. After excluding alternative etiologies including active infection, autoimmune disorders, and acute myocardial infarction, and conducting comprehensive medication review, the association between micafungin and the adverse events was assessed as “Probable” according to Naranjo Scale. Following micafungin discontinuation and supportive hydration therapy, the CK, MYO, and troponin levels normalized synchronously, with marked clinical improvement. No subsequent systemic antifungal agents were administered, with pulmonary infection controlled through active bronchoscopic management. During the six-month post-discharge follow-up until October 2025, she remained clinically stable with no readmissions for recurrent pulmonary infection or similar adverse reactions. Study limitations include inadequate coronary artery visualization on pre-admission CTA (March 19, 2025) due to poor patient cooperation, and inability to perform follow-up coronary CTA, cardiac magnetic resonance imaging (MRI), or muscle biopsy during convalescence due to the patient’s fragile condition, leaving certain pathophysiological questions unresolved.

## 4 Conclusion

This study presents the first documented case of micafungin-induced RM with concomitant myocardial injury, serving as an important clinical alert for high-risk populations receiving this antifungal agent (particularly elderly patients with multiple comorbidities). We strongly recommend close monitoring of cardiac enzymes, troponin levels, urinalysis, and renal function parameters during micafungin therapy, especially when patients develop unexplained myalgia or cardiac symptoms. Further research is warranted to elucidate the precise pathogenic mechanisms underlying micafungin-associated RM and myocardial injury. Clinicians should maintain heightened awareness of this rare but potentially life-threatening adverse reaction to ensure medication safety.

## Data Availability

The original contributions presented in the study are included in the article/supplementary material, further inquiries can be directed to the corresponding author.
